# Ergonomic evaluation of computer workers

**DOI:** 10.47626/1679-4435-2021-628

**Published:** 2021-12-30

**Authors:** Michelli Belotti Bersanetti, Camila Gorla, Aline Mendonça Turci

**Affiliations:** 1 Departamento de Ciências Biológicas e da Saúde, Universidade de Araraquara, Araraquara, SP, Brazil.

**Keywords:** ergonomics, checklist, occupational health, computer, pain, ergonomia, lista de checagem, saúde do trabalhador, computador, dor

## Abstract

**Introduction:**

Information technologies have become indispensable in the office environment with a considerable increase in the use of computers. Musculoskeletal complaints in computer workers have a multifactorial etiology; therefore, an ergonomic investigation should be based on both self-reporting of symptoms and quantifiable observational methods.

**Objectives:**

This study aimed to evaluate ergonomic and biomechanical characteristics of computer workers to identify the presence of symptoms and to assess the existence of a correlation between experts’ observational assessment and workers’ self-perception.

**Methods:**

Participants were approached by an observer responsible for screening of symptoms and demographic characteristics. Volunteers were then evaluated simultaneously by two blinded observers.

**Results:**

Seventy-one computer workers participated, and no significant differences were observed for duration of work on a computer between participants with and without pain. Interobserver reliability was good (0.93, 95%CI 0.88-0.96). No correlation was found between Maastricht Upper Extremity Questionnaire and Rapid Office Strain Assessment scores (p = 0.054/r = 0.230). There was no difference between participants with and without pain in the Rapid Office Strain Assessment (p = 0.931). In the Maastricht Upper Extremity Questionnaire assessment, there were differences in job demand (p = 0.004), complaints (p = 0.034), and total score (p = 0.044), with higher scores for asymptomatic participants.

**Conclusions:**

The results suggest that asymptomatic individuals are subject to higher job demands probably because they have not previously experienced significant pain. However, they have other complaints, such as stiffness, disability, weakness, edema, and paresthesia. Symptomatic individuals, in turn, have greater trouble in aspects such as reduced amount of time spent on work and performance of work requiring extra effort.

## Resumo

**Introdução:**

As tecnologias da informação tornaram-se indispensáveis no ambiente de escritório, resultando no aumento considerável do uso do computador. As queixas musculoesqueléticas em trabalhadores usuários de computador possuem etiologia multifatorial; assim, no campo da ergonomia, a investigação deve ser baseada em relato de sintomas e por métodos observacionais quantificáveis.

**Objetivos:**

Avaliar aspectos ergonômicos e biomecânicos de trabalhadores de escritório usuários de computador, identificar a presença de sintomas e verificar a existência de correlação entre a avaliação observacional e a percepção dos colaboradores.

**Métodos:**

Os participantes foram abordados por um avaliador responsável pela triagem, que incluiu sintomas e características demográficas. Em seguida, o voluntário foi avaliado simultaneamente por dois avaliadores cegados entre si e para as características dos participantes.

**Resultados:**

Participaram 71 trabalhadores, não sendo observadas diferenças significativas para tempo de trabalho entre os indivíduos com e sem dor. A confiabilidade interavaliador foi considerada forte (0,93; IC95% 0,88-0,96). Não foi encontrada correlação entre o Maastricht Upper Extremity Questionnaire e o Rapid Office Strain Assessment (p = 0,054/r = 0,230). Não foi observada diferença entre os indivíduos com e sem dor no Rapid Office Strain Assessment (p = 0,931). Já na análise do Maastricht Upper Extremity Questionnaire, houve diferença nos domínios demanda (p = 0,004), queixas (p = 0,034) e total (p = 0,044), com maior pontuação para os assintomáticos.

**Conclusões:**

Os resultados sugerem que os indivíduos assintomáticos se submetem a uma maior demanda de trabalho, provavelmente devido à ausência de experiências prévias significativas de dor. Entretanto, apresentam outras queixas, com ênfase em rigidez, incapacidade, fraqueza, edema e parestesia. Já os sintomáticos apresentam maior dificuldade em questões como tempo dedicado ao trabalho e execução do tempo de trabalho, necessitando realizar esforços extras.

## INTRODUCTION

Ergonomics is the scientific study of the relationship of people with their work instruments, methods, and environment, aiming to improve human safety and well-being.^1^ Information technologies have become indispensable in the office environment, causing the use of computers as part of work to have increased considerably in recent years.^2^ In 2000, 60% of workers were required to use a computer for their job duties, and 80% reported using it on a daily basis.^3^

Increased use of computers stems from economic development in recent decades, which led to organizations using more computerization systems and devices to improve the productivity of their workers.^4^ In a review conducted by Wahlström^5^ of ergonomic studies on musculoskeletal disorders and their association with computer work, the prevalence of musculoskeletal disorders was 10 to 62%. This demonstrates that increased use of technologies at work has come at the cost of workers’ well-being.

Workplace risk factors that may cause changes in the health of computer workers correspond to the physical demands imposed by performing their activities, including the postures adopted to perform job duties (generally static), the applied force, the duration of tasks, and the frequency and repetition of movements.^6^ There are also psychosocial factors related to work, such as time pressure, lack of social support, and poor job satisfaction, which may contribute to the development of work-related musculoskeletal disorders (WMSDs).^6^

The main musculoskeletal disorders associated with computer work affect the upper extremities,^7^ neck and head,^8^ and spine.^9^ Musculoskeletal complaints among computer workers have a multifactorial etiology,^5,10^ and the main causes include poor postures, bad habits at work, workstation design, and psychosocial factors.^5^ Among these multiple factors, biomechanical (postures, changes in body scheme, protective muscle patterns, and physical unfitness)^11^ and lifestyle (sedentary behavior, inactivity, and sleep debts) characteristics are noteworthy.^12^

In the field of ergonomics, the most used method to investigate the influence of biomechanical, ergonomic, and psychosocial variables on musculoskeletal disorders is based on self-reporting of symptoms.^13^ This technique is advantageous because different aspects are analyzed considering the individual’s self-perception. It is also economically feasible and quick to administer, which makes it a large-scale approach.^14^

The main disadvantages of using self-report instruments are possible inaccuracies and/or unreliability of reports as well as individual differences in the levels of education, understanding, and interpretation.^15^ Thus, an alternative way to provide more reliable data on working conditions is to combine self-report instruments with preferably quantifiable observational methods, such as checklists, which can be completed directly (in loco observation) or indirectly (image recordings).^15^

The Maastricht Upper Extremity Questionnaire (MUEQ)^16^ is one of a few tools in the scientific literature to assess the workers’ self-perception of ergonomic and psychosocial aspects, being specific for computer workers. The Rapid Office Strain Assessment (ROSA), in turn, is a checklist used for assessing postures of computer workers in the office environment.^17^ Unlike the MUEQ tool, the ROSA checklist is intended to assess postures through experts’ observation. ROSA was designed to quantify musculoskeletal risks in different postures, with good levels of sensitivity (76%), specificity (68%), and interobserver reliability.^18^

Researchers have given increasing attention to issues related to workers’ health status and quality of life.^19^ The generally affected health categories are physical, social, and emotional functions as well as self-perceived health and well-being,^20^ which can all be measured by the Medical Outcomes Short-Form Health Survey (SF-36).^21^

A systematic review of cohort studies found limited evidence that irregular body posture, poor computer skills, distance of keyboard from edge of the table less than 15 cm, high task difficulty, low job control, and perceived muscular tension are associated with the development of musculoskeletal pain.^22^ The authors reported a lack of ergonomic studies and the need for an observational assessment of workstations, reinforcing that this should not be based on self-reporting only.^22^

Thus, this study aimed to evaluate ergonomic and biomechanical characteristics of computer office workers at a private institution and to identify the presence of symptoms. It also investigated the degree of correlation between the ROSA observational assessment of biomechanical and ergonomic factors and the MUEQ self-perception assessment of workstations and body postures.

## METHODS

### PROCEDURES

The participants were initially approached by an observer (O1) who was responsible for conducting the screening process containing demographic questions and for distributing the MUEQ-Br^16^ and SF-36^21^ questionnaires. Volunteers who met the eligibility criteria were then visited by two new observers (O2 and O3), blinded to demographic characteristics and symptoms of the participants. These two observers performed an individual but simultaneous assessment of the workstations (ROSA checklist).^17^ Importantly, O2 was not aware of the assessment of O3, and vice versa. The study was approved by the Research Ethics Committee at Universidade de Araraquara (CAAE No. 60154916.6.0000.5383) in accordance with the standards established by the Brazilian National Health Council Resolution No. 466/12. All individuals who agreed to participate signed an informed consent form.

### SAMPLE

The study sample consisted of computer office workers at a private university. They were aged 18 to 50 years, had the same job for at least 12 months, and performed at least 4 hours of computer work per day.^18^

Those who were illiterate, those with a cognitive deficit, visual impairment (not corrected by glasses), hearing loss (not corrected by hearing aids), or diseases causing intellectual impairment, those with a history of fracture, dislocation, and/or surgery in the upper extremities, head, and spine, and those with systemic disorders (fibromyalgia, lupus, or rheumatoid arthritis) were excluded.

For characterization of the participants, a pain numerical rating scale^23^ was used to identify symptomatic participants and to determine pain severity. This is an 11-point scale ranging from 0 (“no pain”) to 10 (“pain as bad as could be”).

In addition, self-perceived health status was assessed with the SF-36,^24^ which is a generic quality-of-life instrument that is easy to understand and administer. It has eight components: physical functioning, limitations due to physical problems, pain, general health perception, vitality, social functioning, limitations due to emotional problems, and mental health. There are 36 items with a total score ranging from 0 to 100 points. This system was developed so that the highest score (100) indicates best health status and 0 corresponds to worst health status.

### ERGONOMIC EVALUATION

The ROSA checklist^17^ and the MUEQ self-report tool^16^ were used to assess ergonomic and biomechanical aspects. These two approaches were compared and, to ensure that the observers’ assessment was reliable, the reproducibility of ROSA was carried out by two blinded observers.

#### Revised MUEQ-Br

The MUEQ^16^ is a tool available for assessment of ergonomic and psychosocial aspects, being specific for computer workers. Also, the tool was developed to characterize in detail complaints of the arm, neck, and shoulder (CANS).^10^ The revised MUEQ-Br has 41 questions divided into six domains: workstation, body posture, job control, job demand, break time, and social support.^16^

The domains are scored as follows: workstation - six questions referring to aims and physical space, with a maximum score of 7 points; body posture - six questions referring to body position, with a maximum score of 18 points; job control - nine questions referring to self-management, with a maximum score of 27 points; job demand - seven questions referring to work pressure, with a maximum score of 21 points; break time - six questions referring to duration of breaks, with a maximum score of 18 points; social support - seven questions referring to work routine, with a maximum score of 21 points. It also includes complaint items to characterize the CANS.^16^

### ROSA

ROSA is an ergonomic checklist designed to quantify exposure to musculoskeletal risk factors in different postures of computer workers in the office environment. ROSA is based on other checklists and aims to identify the degree of ergonomic risk using specific scores. The Brazilian Portuguese version was validated by Rodrigues et al.^17^

ROSA is based on a visual assessment of individuals at their workstations. Thus, the observer will select the observed postures as well as the time spent to perform that task while maintaining a specific posture. ROSA is divided into three sections (A, B, and C) - A assesses posture in the chair, B assesses position of the monitor and telephone, and C assesses position of the keyboard and mouse. Finally, scoring charts are used to calculate a final score ranging from 0 to 10.

Sonne et al.^18^ demonstrated a correlation between higher levels of discomfort and increased ROSA scores. The final score is used to assign the observed posture to an action level, indicating the recommended intervention. The four action levels are: level 1 (scores 1 and 2) - posture is acceptable if not maintained and repeated over a long period; level 2 (scores 3 and 4) - further investigation is needed; level 3 (scores 5 and 6) - further investigation and changes are required soon; and level 4 (scores 7 or over) - investigation and changes are required immediately.

### DATA ANALYSIS

The results obtained were submitted to basic descriptive statistical procedures, and the variables were described with mean, standard deviation (SD), and 95% confidence interval (95%CI). The significance level was set at 0.05. Data distribution was assessed by the Shapiro-Wilk test, and equality of variances, by the Levene test. For data with a normal behavior, the Student t-test was used. For those with a non-normal behavior, the nonparametric Mann-Whitney test was used with a significance level of 0.05.

The correlation of MUEQ workstation domain, body posture domain, and total scores with ROSA total scores was assessed with Spearman rank correlation coefficient (rs). The correlation was rated as strong (rs = 0.70), moderate (rs = 0.40 or rs < 0.70), or weak (rs < 0.40), with a significance level of 0.05.^25^

Intraclass correlation coefficient (ICC) and its respective 95%CI were used for statistical analysis of reproducibility. The ICC is interpreted as follows: less than 0.40 - poor reproducibility; between 0.40 and 0.70 - moderate reproducibility; greater than 0.75 - good reproducibility.^26^

## RESULTS

Seventy-one computer workers participated in this study. No significant differences were observed for anthropometric data and duration of work on a computer between participants with and without pain (Table 1). However, symptomatic participants (5.72; SD 2.19) reported significantly higher pain severity (p = 0.00) than asymptomatic participants (0.0; SD 0). Perceived limitations due to physical (p = 0.021) and social aspects (p = 0.023) and pain (p = 0.001), as assessed by the SF-36, were different between the groups with and without pain (Table 1).

**Table 1:** Characteristics of the total sample divided by presence or absence of symptoms, Araraquara, São Paulo, Brazil, 2020 (n = 71)

All	Asymptomatic	Symptomatic
n = 71	n = 24	n = 47
Age: 35.28 years (12.08)	Age: 32.50 years (11.40)	Age: 36.70 years (12.30)
Weight: 71.76 kg (14.94)	Weight: 67.80 kg (14.50)	Weight: 73.62 kg (14.93)
Height: 1.68 m (0.09)	Height: 1.70 m (0.09)	Height: 1.68 m (0.09)
YWC: 13.38 years (8.98)	YWC: 12.50 years (8.10)	YWC: 13.85 years (9.44)
HWCD: 7.66 hours (0.91)	HWCD: 7.49 hours (0.92)	HWCD: 7.77 hours (0.87)
Physical functioning: 99.65 (2.13)	Physical functioning: 99.58 (2.04)	Physical functioning: 99.68 (2.19)
Limitations due to physical problems: 86.27 (23.83)	Limitations due to physical problems: 93.75 (13.29)	Limitations due to physical problems: 82.45 (27.04)
Pain: 62.59 (24.87)	Pain: 75.88 (25.11)	Pain: 55.81 (22.06)
General health: 61.07 (17.60)	General health: 66.71 (13.60)	General health: 58.19 (18.81)
Vitality: 57.04 (20.26)	Vitality: 62.08 (19.22)	Vitality: 54.47 (20.49)
Social functioning: 72.71 (25.56)	Social functioning: 77.60 (24.16)	Social functioning: 70.21 (26.14)
Limitations due to social aspects: 78.40 (32.42)	Limitations due to social aspects: 88.92 (21.25)	Limitations due social aspects: 73.04 (35.93)
Mental health: 65.69 (19.57)	Mental health: 68.83 (20.53)	Mental health: 64.09 (19.08)
Pain severity: 3.73 (3.24)	Pain severity: 0 (0)	Pain severity: 5.72 (2.19)

Values are shown as mean (standard deviation).

n = sample size; HWCD = hours working on a computer per day; YWC = years working on a computer.

In the reliability analysis, two observers independently but simultaneously assessed the workstations. The interobserver reliability ICC for the ROSA total score was 0.93 (95%CI 0.88-0.96); when the sample was divided by presence or absence of pain, the ICC was 0.91 (95%CI 0.84-0.95) for symptomatic participants and 0.96 (95%CI 0.90-0.98) for asymptomatic participants, demonstrating good reliability.^26^ The correlation analysis between ROSA total scores and MUEQ workstation and body posture domain scores was not significant (Table 2).

**Table 2:** Correlation between Rapid Office Strain Assessment (ROSA) total scores and Maastricht Upper Extremity Questionnaire (MUEQ) workstation and body posture domain scores, Araraquara, São Paulo, Brazil, 2020 (n = 71)

	MUEQ - Workstation domain	MUEQ - Body posture domain	MUEQ - Total score
ROSA Total sample n = 71	r = 0.143 p = 0.236	r = 0.073 p = 0.543	r = 0.230 p = 0.054
ROSA Symptomatic n = 47	r = 0.061 p = 0.690	r = 0.176 p = 0.242	r =-0.039 p = 0.801
ROSA Asymptomatic n = 24	r = 0.207 p = 0.332	r = 0.304 p = 0.148	r = 0.468 p = 0.210

p = significance level < 0.05; r = Spearman rank correlation coefficient.

No difference in ROSA total scores was observed between symptomatic and asymptomatic participants; however, an analysis of MUEQ domain scores showed differences in job demand (p = 0.004), complaints (p = 0.034), and total score (p = 0.044), with higher scores for asymptomatic participants (Table 3). It is worth noting that the ROSA action level (scores 5 and 6) for possible changes in workstations was not achieved; however, there was a need for further investigation.

**Table 3:** Comparison of mean scores for the ergonomic tools used between symptomatic and asymptomatic participants, Araraquara, São Paulo, Brazil, 2020 (n = 71)

	Maximum score (points)	Symptomatic n = 47	Asymptomatic n = 24	p-value
ROSA	10	4.76 (4.30-5.23)	4.87 (4.58-5.15)	0.931
MUEQ				
Workstation	6	1.06 (0.69-2.37)	1.08 (0.61-2.26)	0.951
Body posture	18	6.06 (5.07-6.57)	6.88 (5.74-7.45)	0.329
Job control	27	6.13 (4.59-7.67)	6.21 (4.37-8.05)	0.950
Job demand	21	2.83 (2.04-3.61)	6.58 (4.33-8.84)	0.004*
Break time	18	4.91 (3.82-6.01)	5.25 (4.10-6.40)	0.707
Social support	21	1.70 (0.88-2.53)	1.42 (0.45-2.38)	0.679
Complaints	55^†^	7.09 (5.16-9.01)	11.79 (8.07-15.51)	0.034*
Total score	111/166^†^	35.11 (12.65-40.17)	44.83 (12.64-53.26)	0.044*
Pain severity	10	5.72 (5.09-6.34)	0.000 (0-0)	0.000*

Values are shown as mean and 95% confidence interval (95%CI).

MUEQ = Maastricht Upper Extremity Questionnaire; n = number;

ROSA = Rapid Office Strain Assessment.

* p < 0.05 for Student t-test.

† Maximum total score considering the complaint items.

## DISCUSSION

Based on the characteristics presented by the study sample, how individuals with pain interpreted their limitations due to physical and social aspects differed from that of those without pain. Symptomatic participants reported greater trouble in SF-36 items addressing topics such as cutting down the amount of time spent on work or other activities and having difficulty performing work or other activities and taking extra effort. In line with these findings, concerning the MUEQ job demand domain, which includes questions about working under extensive pressure, working extra hours to finish tasks, and speeding to finish tasks on time, asymptomatic participants were found to be subject to higher job demands.

There was no difference between the groups with and without pain regarding ROSA total scores and MUEQ domain scores, except for job demand and complaints; therefore, the physical aspects of the workstation were not associated with pain. It is assumed that participants without pain are subject to a higher job demand than those with pain, which may have influenced the higher score in the complaint items and, consequently, the MUEQ total score. Importantly, the MUEQ complaint items address not only presence of pain but also other complaints, such as weakness, stiffness, disability, edema, and paresthesia. Also, other authors suggest that these items should only be used to characterize the sample and symptoms, and should not be scored and used for analyses such as the one in the present study.^16^

Contrary to our findings, a study showed that computer workers who reported a lower job demand had pain less frequently in all anatomical areas investigated, except in the lower back; the Rapid Upper Limb Assessment (RULA) score in the workstation observational assessment differed significantly between the groups with and without pain.^2^ Regarding mean scores in the observational assessment of the present study, the action level for workstation interventions was not achieved; however, the score is close to 5, and the CI suggests that the level has been reached, which means that further investigation and changes will be required soon.

Another hypothesis about a lower job demand in individuals with pain is that they tend to be more cautious because of previous experiences of musculoskeletal disorders, which generate feedback influencing the perception of mental stress and job organization.^5,16^ However, a systematic review conducted by Paksaihol et al.^22^ did not confirm that psychosocial factors have a predictive value for upper-extremity complaints.

The correlation analysis included ROSA total scores and MUEQ workstation and body posture domain scores, as they are equivalent constructs; however, they did not correlate, probably because workers and observers had different perceptions. As previously mentioned, self-report tools may be poorly reliable because they are influenced by interpretation.^15^ In this study, the participants, despite having an acceptable level of education, were not experts in the topic.

Also, although workers were asked to maintain the postures they usually adopted during the assessment, these may have been modified, as the analysis of symptomatic and asymptomatic participants showed no statistical difference. Previous studies^27,28^ showed a relationship between musculoskeletal symptoms and poor upper-extremity postures. In contrast, Gawke et al.^29^ analyzed physical aspects of the work environment together with psychosocial factors and found no associations with neck and upper-extremity complaints.

Regarding self-reported pain, the data suggest that it is not caused by the workstation or by occupational factors. Therefore, this supports the idea that factors not controlled for in this study may have contributed to the onset of pain, such as level of physical activity, sleep quality, diet, and other activities, either paid or unpaid, that employees may have engaged in outside the study setting. According to Mattioli et al.,^30^ there is insufficient data in the scientific literature to confirm that computer work is associated with neck and upper-extremity complaints. A study conducted by Turci et al.^27^ to evaluate computer workers at a private institution suggested that symptoms may not be linked to the occupational activity.

A limitation of this study was the sample size; thus, additional studies with larger samples and similar studies addressing other private sector categories for wider data coverage should be conducted. Furthermore, psychosocial aspects such as pain catastrophizing, anxiety and depression, beliefs, fear, and pain avoidance were not controlled for. A strength of this study was the correlation analysis between the MUEQ tool, which shows self-perceptions of computer workers, and the ROSA checklist, which captures ergonomic and biomechanical characteristics of computer workers by observers. The results demonstrated that those viewpoints may be divergent.

## CONCLUSIONS

Asymptomatic individuals are subject to higher job demands probably because they have not previously experienced significant pain, although they have other complaints, such as stiffness, disability, weakness, edema, and paresthesia. Thus, absence of pain does not mean absence of complaints that could compromise occupational health. Symptomatic individuals, in turn, have greater trouble in aspects such as reduced amount of time spent on work and performance of work requiring extra effort. Regarding the findings of the observational assessment, workstation interventions are not required; however, this should be analyzed cautiously, given that further investigations are required, such as anthropometric measurements.
